# Preventing the transmission of American trypanosomiasis and its spread into non-endemic countries

**DOI:** 10.1186/s40249-015-0092-7

**Published:** 2015-12-28

**Authors:** Qin Liu, Xiao-Nong Zhou

**Affiliations:** National Institute of Parasitic Diseases, Chinese Center for Disease Control and Prevention; Key Laboratory of Parasite and Vector Biology, Ministry of Health;, WHO Collaborating Center for Tropical Diseases, Shanghai, 200025 P. R. China

**Keywords:** American trypanosomiasis, Chagas disease, *Trypanosoma cruzi*, Transmission control

## Abstract

American trypanosomiasis, commonly known as Chagas disease, is caused by the flagellate protozoan parasite *Trypanosoma cruzi*. An estimated eight million people infected with *T. cruzi* currently reside in the endemic regions of Latin America. However, as the disease has now been imported into many non-endemic countries outside of Latin America, it has become a global health issue. We reviewed the transmission patterns and current status of disease spread pertaining to American trypanosomiasis at the global level, as well as recent advances in research. Based on an analysis of the gaps in American trypanosomiasis control, we put forward future research priorities that must be implemented to stop the global spread of the disease.

**Electronic supplementary material:**

The online version of this article (doi:10.1186/s40249-015-0092-7) contains supplementary material, which is available to authorized users.

## Multilingual abstracts

Please see Additional file 1 for translations of the abstract into the six official working languages of the United Nations.

## Introduction

American trypanosomiasis, commonly known as Chagas disease, is caused by the hemoflagellate protozoan parasite *Trypanosoma cruzi*. It has been a neglected tropical disease and an important health problem in Latin America for many decades. With no vaccine yet available, only two proven drugs, namely benznidazole and nifurtimox, can be used for efficient treatment of acute cases. However 95 % of untreated patients advance into the chronic stage of the disease; at least 30 % then develop chagasic cardiomyopathy and up to 10 % can develop digestive, neurological, or mixed alterations. These all can lead to high morbidity and mortality rates among adults in endemic countries; the current number of annual deaths is at least 10,000 [1]. Chagas disease has been estimated to cost approximately 667,000 disability-adjusted life years [2]. The World Bank and World Health Organization (WHO) consider Chagas disease as the fourth most important infectious disease after malaria, tuberculosis, and schistosomiasis [3].

**Fig. 1:**
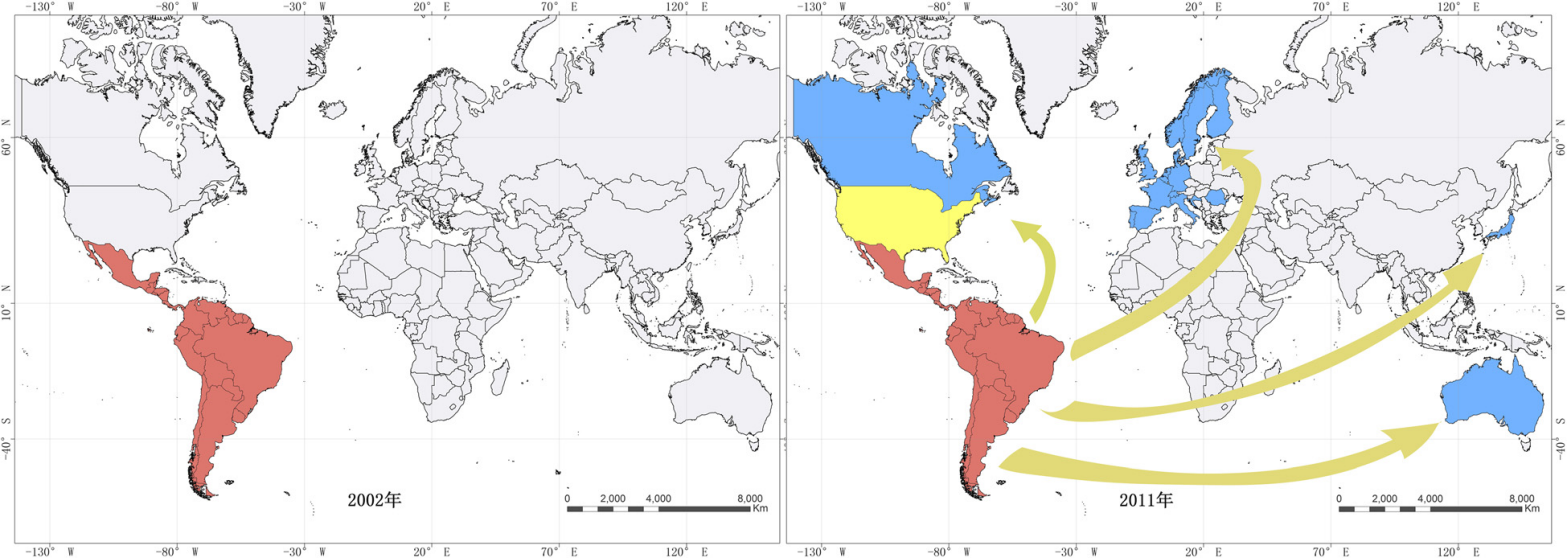
Mapping data showing epidemiological changes pertaining to Chagas disease between 2002 and 2011 (red refers to endemic areas where transmission is through vectors; yellow refers to endemic areas where transmission is occasionally through vectors; blue refers to non-endemic areas where transmission is through blood transfusion or organ transplantation, etc.)

The disease is estimated to affect around eight million people in the Western Hemisphere, who are mainly distributed in Latin America. At least 120 million people are at risk of contracting the disease [1]. The highest prevalence of Chagas disease has been reported in Bolivia (6.75–15.4 %), followed by Paraguay (0.69–9.3 %) and Panama (0.01–9.02 %) [4, 5] (see Table 1). However, the total number of cases in Brazil (0.8–1.30 %), Mexico (0.5–6.8 %), and Argentina (4.13–8.2 %) together account for almost 60 % of all people infected with *T. cruzi* in Latin America [4, 5]. In the last decade, due to increasing levels of migration, important epidemiological changes have occurred and the disease has now spread to non-endemic countries. Geospatial data from 2002 to 2011 demonstrated that Chagas disease has existed in countries outside of Latin America (see Fig. 1). For instance, Chagas disease has been diagnosed in non-endemic countries in North America, such as Canada and the United States (US); in the Western Pacific Region, such as Australia and Japan; as well as in Europe [6–9]. Transmission of Chagas disease has now become a global health issue and has attracted much more attention than ever before [10]. In this review, we outline the research priorities needed to stop the current spreading pattern of the disease based on a gap analysis of needs in the epidemiology and control of American trypanosomiasis.

**Table 1:** Burden of Chagas disease and screening of blood donors for *T. cruzi* in Latin America

Country	Estimated *T. cruzi* prevalence (%) [5]	Transmission by principal vector (year certified) [30]	Coverage of blood-donor screening (%) [29]	Seropositive level of blood donor (%) [29, 75]
Argentina	4.13–8.20	Not interrupted^a^	100	4.50
Belize	0.74	Interrupted	100	0.40
Bolivia	6.75–15.40	Not interrupted	86	9.90
Brazil	0.80–1.30	Not interrupted^a^	100	0.61
Chile	0.99–2.80	Interrupted (1999)	75^b^	0.47
Colombia	0.48–1.20	Not interrupted	99	2.80
Costa Rica	0.53–11.70	Interrupted	99	0.98
Ecuador	0.20–1.74	Not interrupted	100	0.15
El Salvador	3.37–6.10	Interrupted (2010)	100	2.46
Guatemala	1.98–7.89	Interrupted (2009)	100	0.79
Honduras	3.05–5.80	Interrupted (2011)	100	1.40
Mexico	0.50–6.80	Not interrupted^a^	100^c^	1.50
Nicaragua	1.14–1.70	Interrupted (2011)	100	0.49
Panama	0.01–9.02	Not interrupted	98	0.90
Paraguay	0.69–9.30	Not interrupted^a^	99	2.80
Peru	0.20–3.00	Not interrupted^a^	99	0.26
Uruguay	0.60–1.20	Interrupted (1997)	100	0.47
Venezuela	1.16–4.00	Not interrupted	100	0.67

^a^The principal vector has been eliminated in some provinces and transmission by the principal vector has been interrupted in some provinces

^b^98 % in endemic regions

^c^Screening for 18 government-run transfusion centers

## Review

### Patterns of American trypanosomiasis transmission

In endemic areas, the most common way *T. cruzi* infections occur are through vector-borne transmission by triatomine insects, followed by blood transfusions [11]. Triatomine bugs, especially *Triatoma infestans* and *Rhodnius prolixus,* are considered to be the most important two vectors of *T. cruzi*. In non-endemic areas, where no vectors exist, other transmission routes, such as blood transfusions, organ transplantation, or congenital transmission, are predominant.

**Fig. 2:**
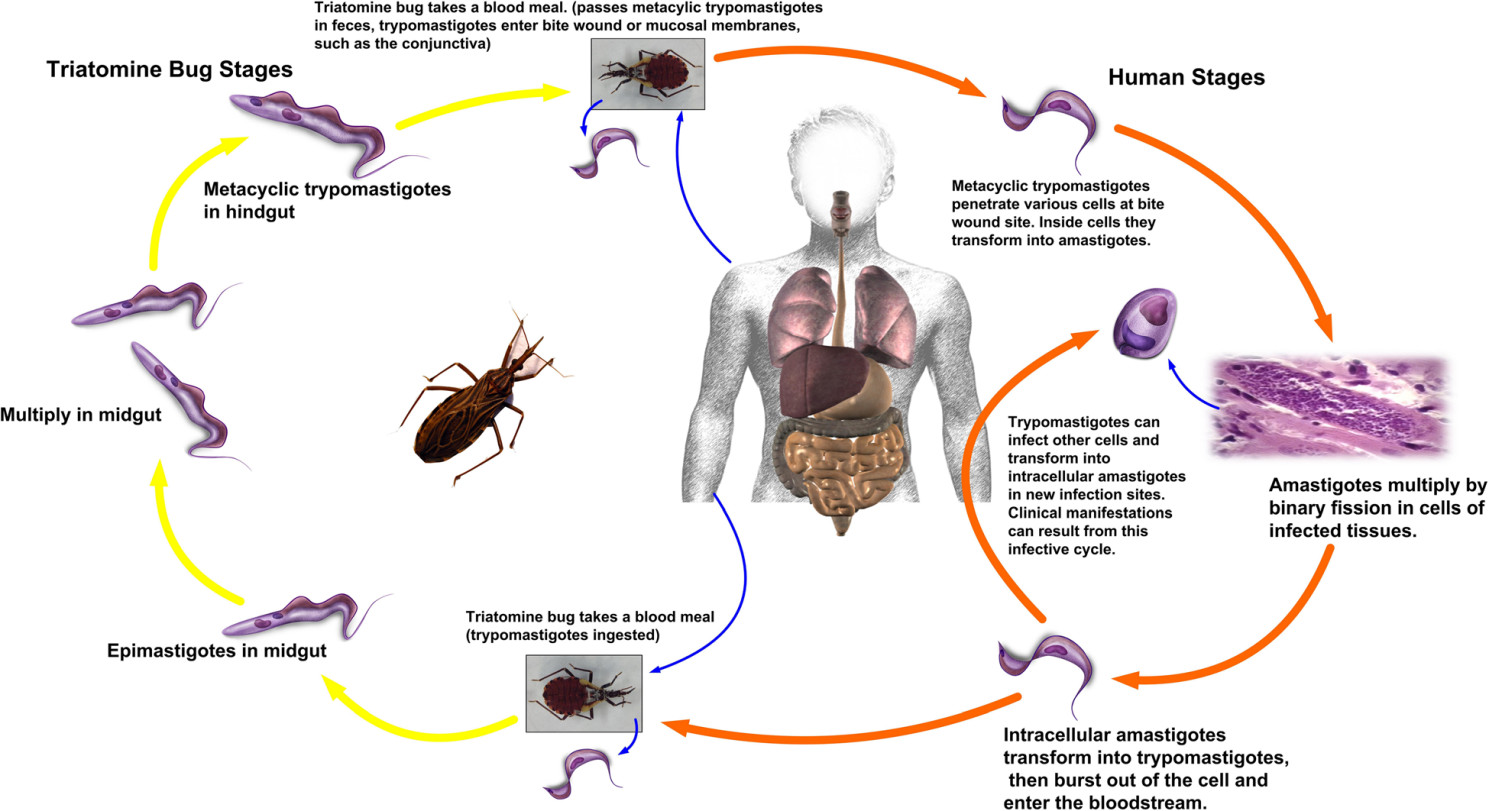
Life cycle of the *T. cruzi* parasite in triatomine insects and humans

Transmission routes of Chagas disease are generally divided into two streams, based on the frequency of transmission and epidemiological importance. Firstly, the most common routes for *T. cruzi* transmission include vector transmission, oral transmission, transfusion, and vertical or congenital transmission [12]. In the vector transmission mode, *T. cruzi* is transmitted by blood-sucking bugs of the family Reduviidae, subfamily Triatominae. This transmission route has the largest impact in Latin American countries and is also responsible for maintaining the pathogen life cycle of the disease. In this case, the parasite develops in the body of the insect vector after it has fed on the blood of an infected host. The infective form of the parasite is then transmitted to humans via the excreta of the triatomine insect through mucous membranes or breaks in the skin. Parasites then enter the bloodstream and invade cells of the monophagocytic system. The life cycle of *T. cruzi* transmitted through triatomine insects is shown in Fig. 2. Although more than 140 different species of triatomines have been identified, only a relatively small number are significant vectors. Fourteen species are the main vectors in the sylvatic and domestic cycle, namely *Triatoma infestans, T. sordida*, *T. pseudomaculata*, *T. tibiamaculata*, *T. arthurneivai*, *T. brasiliensis*, *T. dimidiata*, *Panstrongylus megistus*, *P. geniculatus*, *P. diasi*, *Rhodnius neglectus*, *R. prolixus*, *R. megistus*, and *R. domesticus* [9]. However, the main species related to *T. cruzi* transmission vary according to different regions and settings. For instance, *T. infestans* is more dominant in Southern Cone countries, whereas *R. prolixus* is closely associated with palm trees and maintains enzootic *T. cruzi* cycles in Central America [3, 9]. Triatomines can be of both sexes and have five nymphal stages, all of which are involved in the transmission of *T. cruzi* [3]. Furthermore, *T. cruzi* can be orally transmitted by ingestion of contaminated food or liquid. Several outbreaks of oral Chagas disease have been reported in Brazil, Colombia, and Venezuela, with acute cases arising after patients ate food contaminated with vector feces and urine [13, 14]. The largest worldwide outbreak of oral Chagas disease occurred in Venezuela in 2007, with a total of 103 acute cases, 80 % of those being children [13].

*T. cruzi* can also be transmitted to humans via non-vector mechanisms including blood transfusion and congenital transmission, which are the main causes of infection in urban areas and non-endemic countries. For instance, in Brazil alone, in the 1970s, it has been estimated that 100,000 new Chagas disease cases annually occurred due to blood transfusions [9]. A total of 885,187 blood samples collected in El Salvador between 2001 and 2011 revealed 21,693 cases of transfusion-related infections [15]. There is also evidence of the disease occurring due to blood transfusions in non-endemic countries. Up until 2013, at least eight cases of blood transfusion-related infections were reported in Canada and the US [16]. Congenital or vertical transmission occurs when infected mothers transmit the parasite, which can cross the placental barrier to their offspring during the gestation period [17]. Up until 2014, eleven cases of congenital Chagas disease have been reported in Japan and Europe [18]. A systematic literature review revealed that the estimated global rate of *T. cruzi* congenital transmission was 4.7 %- 5.0 % in endemic countries and 2.7 % in non-endemic countries [19, 20]. The number of annual cases of congenital Chagas disease has been estimated at 14,385 in Latin America, 66–638 in the US, and 20–183 in Europe [17].

Secondly, there are more uncommon or accidental modes of transmission. These include organ transplantation routes, ingestion of maternal milk contaminated with the protozoan parasites, and laboratory accidents [12]. Several cases of transmission via organ transplants have been reported recently as a novel transmission mode of Chagas disease [21, 22]. In the US, it was estimated that approximately 300 patients annually may have acquired Chagas disease through transplants [23]. Furthermore, *T. cruzi* can be orally transmitted by ingestion of contaminated maternal milk. Transmission of Chagas disease from mother to child through contaminated breast milk was first described by Mazza et al. in 1936, but since then, transmission through breastfeeding in humans has not been reported again [17]. Because of this, most scientists and doctors still recommend that breastfeeding is the ideal way of providing nutrition during the first six months of life by mothers with chronic Chagas disease [20, 24]. More rarely, *T. cruzi* can be transmitted to people who work with cultured parasites [25].

### Advances in the control of American trypanosomiasis

Various studies on the interruption of transmission of American trypanosomiasis have been conducted in Latin American countries since the last century, and the progress of these initiatives has been widely reviewed [26, 27]. Since July 1991, multinational initiatives against Chagas disease have been launched in the Southern Cone by the Ministries of Health of Argentina, Bolivia, Brazil, Chile, Paraguay, and Uruguay. In 1997, the initiatives of the Andean Countries, and of Central America and Mexico have been created. All the initiatives have the same or similar aims, which were to (i) interrupt the transmission of Chagas disease by eliminating domestic vectors, (ii) screen blood donors to reduce the risk of transfusion transmission, and (iii) promote maternal screening for infection, followed by treatment of infected newborns where necessary [28, 29]. All endemic countries have joined the multinational initiatives against Chagas disease, namely surveillance and control of the disease and its vectors, at the beginning of this century. Several achievements were implicit to leading to the control of the disease. First, the distribution of domestic vectors has been markedly reduced, and although transmission has not been completely interrupted, effective control measures have been implemented over vast areas. Transmission by principal domestic vectors has been effectively controlled in Uruguay (1997), Chile (1999), Brazil (2006), El Salvador (2010), and substantial areas of Argentina, Bolivia and parts of Central America (see Table 1) [2, 29–31]. Effective vector control has also reduced the reinfection rate of the disease, which was considered a key factor for severe morbidity in earlier decades [32, 33]. Second, extensive screening of blood donors for *T. cruzi* infection has been carried out in most Latin American countries, with the majority having a 100 % coverage rate of blood-donor screening (see Table 1) [2, 12]. It has been reported that the seroprevalence of Chagas disease among blood donors has dropped from 7 to 0.6 % between 1970 and 2006 [12, 34]. The number of people who are infected with Chagas disease has reduced from thirty million people in 1990 to eight million people [1, 2]. This is based on the fact that the new infection rate of Chagas disease has declined to zero in substantial areas in Latin America [2]. Third, congenital infection has been reduced as the apparent rate of transplacental transmission from chronically infected mothers has declined in areas where vector transmission has been interrupted. Consequently, the medical benefits of control initiatives are reflected in the decline of new acute infections and severity of chronic disease cases [29].

With the disease prevalence reduced significantly, a new International Initiative for Chagas Disease Surveillance and Prevention in the Amazon (AMCHA), sponsored by the European Community and the Latin America Triatominae Network, was launched in 2002. This programme has three main objectives, namely to (i) evaluate the risks of Chagas disease in these regions and propose monitoring and prevention of disease transmission, (ii) identify the research requirements for the monitoring and prevention of Chagas disease and, (iii) promote or establish an international cooperation system for the disease [28]. To fulfill this goal, it has been suggested that to best implement disease control, technicians should be trained to recognize triatomines for epidemiology surveillance, in order to make clinical diagnoses and provide treatment. Since then, a Technician Capacitation Manual for the detection of *T. cruzi* has been created [28].

## What needs to be done to control and eliminate the transmission of American trypanosomiasis

### In endemic regions

Although there have been many achievements related to Chagas disease in endemic countries, controlling the disease still poses many challenges due to its zoonotic nature, which involves different domestic and wild reservoirs. Some of the reasons why controlling the transmission of the disease is so difficult are: native vectors reinfesting human habitats, modifications in population dynamics, and the development of resistance to insecticides. The other possible distracting element is that, in some areas, another species of triatomine bugs has adapted or substituted the principal vectors. For example, *T. dimidiata* and *R. ecuadoriensis* may be feasible candidates for elimination in Ecuador and northern Peru, *T. barberi* may be a candidate for eradication in Mexico, and *T. pallidipennis* may require a more long-term control approach in Central America [32]. More recently, the goal for the certification of the “control of the vector transmission of *T. cruzi*” as an alternative to the “interruption of the vector transmission of *T. cruzi*” in endemic countries was proposed by the Southern Cone Initiative to Control/Eliminate Chagas Disease (INCOSUR) [35–37]. Thus, new fundamental concepts have been implicated in the following ways. First is that native vectors has not been eliminable, the entomological surveillance and response system must be sustained, and operational interventions must be continued to minimize infection risks. Second is that there is a need to reestablish a regional evaluation mechanism for the control of native species, as the existing framework was developed on the basis of experiences of controlling vectors and on the sustainability of those control efforts. Some researchers have indicated that they prefer to sustain the surveillance and response systems for native vectors and to certify good practices that can improve disease control [35, 37, 38]. The control of native vectors of Chagas disease is an enduring challenge, and subregional initiatives can greatly contribute to the countries’ efforts by providing result- and process-driven evaluations.

To control Chagas disease, interruption of transfusion transmission is a very important target in control programmes of endemic countries. Therefore, extensive blood-donor screening for *T. cruzi* infection is the main measure implemented in all of the endemic Latin American countries, though the coverage has not yet reached 100 % (see Table 1). After the implementation of the transmission control programme, the rate of new infections has markedly declined over large areas. However, despite this decline, a great challenge still remains. The interruption is defined as seroprevalence being zero among children aged between 0 and 15 years (optionally the target age could be between 0 and 5 years) [35]. Thus, the evaluation requires (i) seroprevalence among children under 15 years of age as the principal indicator, and (ii) geographic coverage of surveillance on native vectors and acute cases [35]. Making the interruption of transmission a regional goal would continue offering political incentives and provide the long-term vision in Latin American countries. Meanwhile, seroprevalence among women of childbearing age (15–44 years of age) has also become a focus because by interrupting congenital or vertical transmission, infections among young individuals can be reduced [39]. There is no reliable method for preventing congenital infection. The most effective strategy is widespread treatment of *T. cruzi*-affected women of childbearing age with routine serological screening, as well as prompt treatment of children born to infected mothers. Information, education, and communication programmes on Chagas disease and its congenital transmission route still need to be strengthened at the community level [20].

Oral transmission of *T. cruzi* through infection by bites of blood-sucking triatomine bugs has aroused attention because of several outbreaks in Latin American countries. This transmission mode is considered an emerging threat because the outbreaks are sporadic, difficult to predict, characterized by high mortality rates, and have shown no signs of declining in frequency or severity [40]. Another challenge is high therapeutic failure, which has been detected in these outbreaks, and data suggest that genetic polymorphism exists in parasite populations [11]. Therefore, the current aim to deal with oral transmission of Chagas disease is focused on identifying the pertinent risk factors, triatomine species involved, and parasite polymorphisms. There is also a need for ongoing epidemiological surveillance and control policies.

Therefore, in endemic countries, long-term regional goals taking into consideration the available evaluation systems to interrupt transmission via intradomiciliary vectors and blood transfusions provide clear direction and incentive to governments, scientific communities, and donors alike.

### In non-endemic regions

Globalization has led to some infectious diseases being widely distributed around the world. Recent trends in global migrations from rural to urban areas, and from endemic to non-endemic countries, have increased the threat of spreading Chagas disease at the global level. For instance, it is estimated that there are more than 26 million Latin American immigrants living in Europe, the US, Canada, Japan, and Australia, which increases the risk of Chagas disease spreading to these non-endemic countries (see Table 2). The first case of imported *T. cruzi* infection was reported in Romania in 1981 [41]. Since then, an increasing number of cases has been reported in Japan, the US, and Europe, which is due to the migration of people from endemic areas [6, 16, 42, 43]. The US has the largest number of Latin American immigrants, estimated at 22 million, with *T. cruzi* infection cases estimated at around 300,167 [44]. In Europe, the estimated total number of migrants from Latin America is more than 3.5 million, with *T. cruzi* infection cases estimated to be between 77,000 and 100,000 [45]. In Canada, Australia, and Japan, there are an estimated total of 100,000, 116,430, and 371,700 Latin American migrants, respectively, with *T. cruzi* infection cases estimated at around 1,789, 1,928, and 3,592, respectively [30, 40, 46, 47] (see Table 2). Due to the absence of vectors in non-endemic countries, prevention and control measures of Chagas disease in such areas must be different from those in endemic areas. The focus should be placed on the risk of transmission through blood transfusions; organ, tissue, or cell transplants; and congenital transmission, as well as potentially acquiring infection during travel to endemic areas. Therefore, the following strategies for prevention of the disease spreading to non-endemic countries are needed.

**Table 2:** Data on American trypanosomiasis spreading to non-endemic countries outside of Latin America*

Indicators	Austria	Belgium	Croatia	Denmark	France	Germany	Italy	Netherlands	Portugal	Romania	Spain	Sweden	Switzerland	UK	Canada	US	Australia	Japan
Estimated number of Latin American immigrants	7552	38,133	ND	ND	208,395	58,000	440,000	35,211	83,000	ND	2,090,695	58,196	60,000–90,000	400,000	100,000	22,000,000	116,430	371,700
Estimated number of *T. cruzi* cases	140–180	1982	ND	ND	2116	935	5,520–7,081	480	850	ND	47,738–67,423	1,118	3,000	14,000	1,789	300,167	1,928	3,592
Number of laboratory confirmed cases	2	19	1	1	111	2	114	7	8	1	3,617	1	256	28	1	799	1	46
Estimated number of pregnant women with *T. cruzi* infection	ND	16	ND	ND	ND	ND	30	18	50	ND	914–1,656	ND	30	50	ND	ND	ND	ND
Estimated number of cases of congenital transmission	ND	1	ND	ND	19	ND	2	2	2	ND	41–121	1	5	5	ND	ND	ND	ND
Number of patients treated +	2	3	ND	ND	28	ND	22	ND	ND	ND	195	1	99	0	ND	ND	ND	ND
Serological screening of blood donors	Yes	No	No	No	Yes	No	Yes	No	Yes	No	Yes	No	Yes	Yes	No	Yes	No	No
Presence of a systematic detection system for congenital infection	No	No	No	No	No	No	Yes^c^	No	No	No	Yes^c^	No	Yes^c^	No^b^	No	No	No	No
Presence of a systematic detection system for organ, tissue, and cell donors of *T. cruzi*	No	No	No	No	Yes	No	Yes	Yes^a^	No	No	Yes	NO	No	Yes	No	No	No	No

* Data sourced from [29, 44–46, 49]

+ Treated with benznidazole or nifurtimox

*ND* Not determined

^a^Only known Chagas disease patients are excluded from donating blood or organs, tissues, and cells for transplantation

^b^Advocacy is carried out for screening of at-risk mothers

^c^Only in some regions or communities

First, more joint efforts should be made at the global level to stop the disease spreading. Since 2007, the WHO has conducted a series of meetings, including “Control and prevention of Chagas disease in Europe (2009)” and “Informal Consultation on Chagas Disease in the Western Pacific (2011),” and issued the World Health Assembling resolution of “WHA63.20 Chagas disease: control and elimination (2010)". The general aim is to control Chagas disease in non-endemic countries and contribute to global efforts to interrupting disease transmission by (i) preventing *T. cruzi* transmission by systematically screening blood used for transfusions and organs intended for transplantation; (ii) clinic diagnosis, case management, and treating patients, including infected newborns through congenital transmission; and (iii) sharing information about Chagas disease, and training health personnel to facilitate diagnosis and medical care [48].

Second, a system for serological screening of blood or tissue donors needs to be established. The United Kingdom (UK) was the first country to implement the systematic screening of at-risk blood donations for *T. cruzi* infection, which has been carried out since 1999. Since then, mandatory screening of blood donors at risk for *T. cruzi* infection has been implemented in Spain (2005), Italy (2005), France (2009), and Switzerland (2013) [48]. In the US, widespread serological screening for blood donations has been initiated, and now covers 75–90 % of blood donors, but screening for *T. cruzi* has not been mandated and remains voluntary [49]. In Sweden, Australia, and Portugal, all individuals at risk for *T. cruzi* infection are excluded from giving blood [48]. In other non-endemic countries, no data has been determined on blood-donor screening until now (see Table 2). However, in Europe, EU guidelines for the quality and safety of blood, tissue, and cell donation are currently being approved. Meanwhile, Italy, UK, Spain, and France have applied measures to identify and detect at-risk donors for organ, tissue, and cell transplantation [48]. In the Netherlands, Chagas disease patients are excluded from donating blood, or organs, tissues and cells for transplantation [50]. In other non-endemic countries, screening of solid organs and tissue is not yet implemented due to a lack of correct techniques being developed. Globally, as part of the systematic detection of congenital infections, there is no legislation requiring the screening of pregnant women for Chagas disease in place, except in some areas of Spain (Catalonia, Galicia, and Valencia) and Italy (Tuscany) (see Table 2). On the other hand, some nongovernmental programmes have being implemented in other regions of Spain and Italy, as well as in Portugal, Switzerland, the US and UK [48].

Third, a surveillance system for Chagas disease supported by prompt detection tests is urgently needed. The surveillance system for Chagas disease has been established at both the national and regional levels in European countries with the coordination of the WHO. However, biological diagnosis is not standardized in terms of the choice of tests and algorithms, and no practical gold standard diagnostics are available. It is recommended that commercial tests become available in non-endemic regions. Frequently, the use of only one test may be sufficient for screening blood donors, but the WHO recommends using two different serological tests for disease diagnosis. The main issue is to screen and treat chronically infected non-pregnant women of childbearing age in order to prevent vertical transmission.

In general, all of non-endemic countries need to work together to increase networking at the global level, which focuses on strengthening global epidemiological surveillance and the sharing of information; prevent transmission by blood transfusions, organ transplantation, and congenital transmission; and promote diagnostic tools for the management of diagnosed cases.

## Research priorities for the control of American trypanosomiasis

Given that more than 100 species of domestic and wild mammals serve as reservoirs for the transmission of Chagas disease, and a number of species of Triatominae serve as potential vectors in nature, this has made the control Chagas disease transmission very difficult. Despite the remarkable progress made by the aforementioned control initiatives, key challenges still need to be addressed.

### Sustainability of control successes

Further efforts are required to maintain and consolidate the achievements made in Chagas disease control, particularly in endemic areas where prevalence is at a low level, and in non-endemic areas where Chagas disease is a new public health issue. Research priorities must be diversified to simultaneously support the development of multiple, alternative control strategies that adapt to these new epidemiological scenarios. In endemic countries, although experience from campaigns against Triatominae throughout the Latin Americas should have led to the main species of domestic populations being interrupted, transmission data from some areas pertaining to peridomestic and wild vectors, rather than domestic ones, is still not available. It is in these areas where disease emergence has been reported and may include local oral outbreaks of food-borne Chagas disease. There have also been reports on the emergence of some resistance foci to pyrethroid insecticides of the main domestic vector, e.g. *T. infestans*, as contributing to the ongoing active transmission of Chagas disease in the Bolivian Chaco region in spite of progress in vector control. In such a scenario, vector monitoring is still a priority.

The development of innovative strategies to explore the biological and behavioral traits of triatomine bugs and paying more attention to new triatomine species is a way to sustain the control programme. This is an important approach for the control of Chagas disease transmission. At the global level, there is a continual need to support established community-based surveillance networks and develop new community networks in disease emerging regions. In order to sustain control efforts, information sharing is of great importance. There needs to be a better integration of knowledge and disciplines into disease prevention and control programmes that goes beyond the involvement of the academic sector, private industry, ministries of government, and local communities. The development of a national database for epidemiological surveillance of Chagas disease using geographical information system (GIS) techniques has also been proposed [29]. The UNICEF/UNDP/World Bank/ WHO Special Programme for Research and Training in Tropical Diseases (TDR) has been working together with the WHO’s information department to establish a global network for a general health surveillance system for Chagas disease. Like other vector-borne diseases, mandatory reporting of all new cases of infection and all findings of domestic, peridomestic, and silvatic populations of Triatominae is a future goal [29].

### Diagnostic tools

The diagnosis of Chagas disease is an important component of national control programmes. Cure rates with early treatment with benznidazole or nifurtimox in infants is close to 100 % and 60–80 % in children under 15 years old, but declines to nearly 0 % in the late chronic phase of the disease [51–53]. It is reported that in Europe, 93.9–96.4 % of cases are underdiagnosed [45]. Therefore, timely diagnosis is very important in order to increase the cure rate. In general, diagnosis of acute infections is based on parasite detection by parasitological studies of direct visualization of *T. cruzi*, whereas diagnosis of chronic American trypanosomiasis relies on serological methods. Serological methods include indirect hemagglutination, indirect immunofluorescence antibody, and enzyme-linked immunosorbent assay (ELISA). Up until now, 11 commercialized *T. cruzi* infection rapid diagnostic tests have been evaluated, with the SERODIA®-Chagas test, the ImmunoComb® II Chagas Ab Kit, and the SD Bioline Chagas Ab Rapid test showing higher performance rates than the other tests [52]. Molecular diagnostic assays have been developed to detect low levels of kinetoplast DNA in the blood of *T. cruzi*-infected individuals. Another scholar has evaluated five serological methods and two molecular methods by rapid diagnostic tests for Chagas disease and proposed that the commercial Bioelisa Chagas® test showed the highest sensitivity and specificity; the amplification of *T. cruzi* DNA in blood samples showed low values of sensitivity, but high values of specificity [54]. Rapid diagnosis of Chagas disease is still a huge challenge because of the high incidence of inconclusive serological reactions and no sensitivity validation for polymerase chain reaction (PCR). It is estimated that the rate of underdiagnosis of Chagas disease in the world is over 94 % [30]. Therefore, research on the diagnosis of Chagas disease is still ongoing. Firstly, genome-wide screening and identification of new *T. cruzi* antigens is being conducted [55]. Secondly, some new techniques have entered clinical trials. These include real-time PCR, which has been proven to be more sensitive and less time-consuming, and may be used to identify highly infectious hosts and implement novel control strategies [56]. Thirdly, the Western blot technique with excretory-secretory antigens of *T. cruzi* epimastigotes has also shown to be effective in the diagnosis of Chagas disease, and it has been reported that this technique can be used as a confirmatory test [57]. A proof-of-concept study of a Chagas urine nanoparticle test (Chunap) also showed good sensitivity to capturing the antigen of *T. cruzi* from urine and was useful for the early diagnosis of congenital Chagas disease [58]. Meanwhile, the applicability of detection techniques in post-therapeutic monitoring of Chagas disease is also essential to understanding the effectiveness of treatment.

### New drugs and treatment

Current treatment options for Chagas disease are limited to only two drugs: benznidazole (Rochagan/LAFEPE and Abarax/ELEA) and nifurtimox (LAMPIT/Bayer). Often, these two drugs are effective in treating acute cases, but show limited efficacy in chronically infected patients. Most people infected with the *T. cruzi* parasite will not develop major clinical manifestations, and only a third of these infections will develop into the chronic phase [59]. Even in the acute stage, a high therapeutic failure detected with differences in drug efficiency ranging from 0 to 100 % has been reported [11]. Treatment with either benznidazole or nifurtimox can present a number of clinical and therapeutic challenges, such as low effective for chronic phase, drug-resistance, etc. As a consequence, there is an urgent need for new safe and efficacious drug treatments that can be applicable both in the acute and chronic phases of the disease. Efforts to deliver new drug candidates for Chagas disease have been very limited. A systematic literature review showed that only naphthoquinones, diamidines, nitroimidazoles and related compounds, and ruthenium complexes have been studied in laboratory settings with failed results due to toxicity or drug-ability issues [60–62]. Recently, many institutions have joined the effort for the discovery of treatment for Chagas disease due to the high-throughput format of screening compounds and the proof-of-concept investigations for identifying the chemical molecular. A typical example of this is the oxaborole series, which has shown potential to treat Chagas disease [63, 64]. In the meantime, two candidate compounds for anti-*T. cruzi*, namely posaconazole and ravuconazole, have been entered into controlled proof-of-concept studies [65]. Unfortunately, the experimental outcome reported a failure due to the long treatment duration (60 days) and no superiority was shown to the old drug, benznidazole. Despite the progress in the identification of new drug candidates to treat Chagas disease, the therapeutic proposal has remained the same for more than 40 years, with no new drug for the treatment of chronic Chagas disease being developed. Although there is still, without a doubt, a knowledge gap, such as the lack of translation between preclinical data and clinical outcomes, drug discovery for Chagas disease is entering a new and exciting era with the development of advanced technology and the drug screening platform [66]. The new data and acquired knowledge could encourage a revision of the target product profile for Chagas disease [66].

### Coinfection and comorbidities

With the interruption of Chagas disease and rapid urbanization, smaller numbers of new cases and acute cases are being reported. Most cases of Chagas disease are now in the chronic stage and frequently turn into a life-long infection. Therefore, awareness on the number of coinfections between mixed infections of different *T. cruzi* genotypes and coinfection with HIV/AIDS has been raised. For example, different pathologies of Chagas disease are caused by six discrete typing units of *T. cruzi* (TcI- TcVI) [67]. Variability of mixed genotypes of *T. cruzi* congenital infection in Chile has been reported, and TcII and TcV lineages of *T. cruzi* were the most frequent in mixed infections [68]. Coinfection with Chagas disease and HIV/AIDS has been reported widely in Brazil, Argentina, and Colombia [68–72]. Out of a total of nine million deaths recorded from 1999 to 2007 in Brazil, coinfection with Chagas disease and HIV/AIDS was mentioned in 74 cases [73]. Polyparasitism or coinfections are more often responsible for high morbidity and mortality rates, and contribute to the higher degree of variability of both disease progression and the success of therapeutic interventions. Early diagnosis, therapy, and monitoring are useful for avoiding reactivations and improving late visceral involvement. For instance, Brazil has established a network for attending to and studying *T. cruzi*/HIV coinfection, and this will be extended to form a Latin American network in the future [73]. Scientists in Spain have provided guidelines for the diagnosis, treatment, and prevention of coinfections in areas where Chagas disease is not endemic. Thorough understanding of the mechanism of polyparasitism and coinfections with Chagas disease is required to improve the likelihood of diagnosis, treatment, prevention, and eventual elimination of Chagas disease [71, 74].

## Conclusion

It has been demonstrated that the transmission of American trypanosomiasis, commonly known as Chagas disease, can be effectively interrupted by controlling the main vectors in endemic areas, which has been implemented in many Latin American countries. Other initiatives to control disease transmission have also been implemented, with great success, to protect the approximately eight million people infected with *T. cruzi* in the continent. However, more recently, Chagas disease has become a global health issue as it has started spreading to non-endemic countries. With globalization and climate change, much more attention has been paid to the spreading of American trypanosomiasis at the global level. More initiatives, such as serological screening of blood donors and surveillance systems to respond to imported cases of Chagas disease, have been established in European and other non-endemic countries.

To prevent the introduction of the disease into non-endemic countries, three main actions are needed: (i) making more joint efforts at the global level to stop the disease spreading, (ii) establishing a system for the serological screening of blood or tissue donors, and (iii) forming a surveillance system for Chagas disease supported by prompt detection tests. In order to stop the current global spreading of the disease, research priorities should focus on the development of (i) innovative strategies to sustain control successes, (ii) new and sensitive diagnostic tools, (iii) new drugs and chemotherapy schemes, and (iv) determinants for measuring coinfection and comorbidities, as well as polyparasitism. These activities will all support Chagas disease control and elimination programmes.

## Additional file

Additional file 1:
**Multilingual abstracts in the six official working languages of the United Nations.** (PDF 207 kb)

## Abbreviations

AMCHA: International Initiative for Chagas Disease Surveillance and Prevention in the Amazon
GIS: Geographical information system
HIV/AIDS: Human immunodeficiency virus infection/acquired immune deficiency syndrome
ELISA: Enzyme-linked immunosorbent assay
INCOSUR: Southern Cone Initiative to Control/Eliminate Chagas Disease
PCR: Polymerase chain reaction
TDR: Special Programme for Research and Training in Tropical Diseases
UK: United Kingdom
US: United States of America
WHO: World Health Organization
