# Supplementary material for: Preventing the transmission of American trypanosomiasis and its spread into non-endemic countries
Source: Infect Dis Poverty. 2015 Dec 28;4:60. doi: 10.1186/s40249-015-0092-7 (PMC4693433; doi:10.1186/s40249-015-0092-7)
Supplement: Additional file 1: — Multilingual abstracts in the six official working languages of the United Nations. (PDF 207 kb) [file 40249_2015_92_MOESM1_ESM.pdf]

Translation of the abstract into the six official working languages of the United Nations

منع انتقال مرض المثقبيات الأمريكي وانتشاره في الدول غير الموبوءة

تشين ليو، شياو نونج زو

ملخص

داء المثقبيات الأمريكي، المعروف باسم مرض شاجاس، يسببه طفيلي وحيد الخلية سوطي يسمى *Trypanosoma cruzi*. هناك ما يقدر بنحو ثمانية ملايين شخص مصابين بـ*T. cruzi* يقيمون حاليا في المناطق الموبوءة في أمريكا اللاتينية. إلا أن المرض انتقل حاليا إلى العديد من الدول غير الموبوءة خارج أمريكا اللاتينية، وأصبح مشكلة صحية عالمية. استعرضنا أنماط انتقال المرض والوضع الحالي لانتشار مرض المثقبيات الأمريكي على الصعيد العالمي، وكذلك التطورات الحديثة في مجال البحوث. استنادا إلى تحليل مواطن القصور في مكافحة مرض المثقبيات الأمريكي، طرحنا أولويات البحوث المستقبلية التي يجب أن تنفذ لوقف الانتشار العالمي لهذا المرض.

Translated from English version into Arabic by Mahmoud Sami, through

TRANSLATORS
WITHOUT BORDERS

美洲锥虫病传播及其在非流行区国家扩散的预防

刘琴，周晓农

摘要

美洲锥虫病，俗称恰加斯病，是由鞭毛体原虫克氏锥虫寄生引起一种热带寄生虫病。目前，估计居住在拉丁美洲的流行区有八百万人感染克氏锥虫。然而，由于这种疾病已被输入到拉丁美洲以外许多非流行区国家，它已成为一个全球性的健康问题。在此，我们回顾了美洲锥虫病传播模式和在全球传播的现状，以及研究进展。基于对美洲锥虫病控制的缺口分析，提出了今后的研究重点是必须实施措施阻止该疾病的全球扩散。

Translated from English version into Chinese by Liu Qin

Prévention de la transmission de la trypanosomiase américaine et de sa propagation dans les pays non endémiques

Qin LIU, Xiao-Nong ZHOU

Résumé

La trypanosomiase américaine, ou maladie de Chagas, est causée par le protozoaire flagellé parasite *Trypanosoma*

cruzi. On estime à huit millions le nombre d'habitants des régions d'endémie d'Amérique latine infectés par *T. cruzi*. La trypanosomiase a cependant été importée dans de nombreux pays non endémiques en dehors du continent sud-américain, pour devenir finalement un problème de santé mondial. Nous avons examiné les schémas de transmission et l'état actuel de propagation de la trypanosomiase américaine au niveau mondial et les récents progrès de la recherche. En nous fondant sur l'analyse des lacunes dans la lutte contre la trypanosomiase en Amérique, nous proposons des priorités pour les recherches futures qui seront nécessaires pour enrayer la propagation mondiale de la maladie.

Translated from English version into French by Suzanne Assenat, through

Предотвращение передачи американского трипаносомоза и его распространения в нехарактерных для данного заболевания странах

Квин ЛИУ, Ксиао-Нонг ДЖОУ

Краткое описание

Возбудителем американского трипаносомоза, или болезни Шагаса, является простейший жгутиковый паразит *Trypanosoma cruzi*. По примерным подсчетам, общее число зараженных данным заболеванием составляет восемь миллионов человек, которые в данный момент проживают в характерных для заболевания регионах Латинской Америки. Однако, на сегодняшний день заболевание было занесено во многие нехарактерные для него страны за пределами Латинской Америки, что стало серьезной причиной для беспокойства среди работников здравоохранения. Мы изучили пути передачи инфекции и текущий статус распространения американского трипаносомоза на международном уровне, а также последние научные разработки. Взяв за основу анализ пробелов в контроле распространения американского трипаносомоза, мы изложили приоритеты для будущих исследований, которые должны быть проведены с целью предотвращения глобального распространения заболевания.

Translated from English version into Russian by Irina Zayonchkovskaya, through

Cómo prevenir la transmisión de la tripanosomiasis americana y su difusión a países no-endémicos

Qin LIU, Xiao-Nong ZHOU

Resumen

La tripanosomiasis americana, comúnmente conocida como Mal de Chagas, es producida por un parásito protozoo flagelado, el *Trypanosoma cruzi*. Se estima que existen ocho millones de personas infectadas con *T. cruzi* que actualmente residen en las regiones endémicas de América Latina. Sin embargo, como la enfermedad ahora ha sido importada por muchos países no endémicos fuera de América Latina, ha pasado a ser un problema de salud mundial. Analizamos los patrones de transmisión y el estado actual de propagación de la tripanosomiasis americana a nivel mundial, así como los avances de investigación recientes. Con fundamento en el análisis de la brechas de control de la tripanosomiasis americana, hemos establecido futuras prioridades de investigación que deben implementarse para detener la propagación de esta enfermedad a nivel mundial.

Translated from English version into Spanish by Maria Alejandra Aguada, through

TRANSLATORS
WITHOUT BORDERS
